# Suprapubic bladder drainage and epidural catheters following abdominal surgery—A risk for urinary tract infections?

**DOI:** 10.1371/journal.pone.0209825

**Published:** 2019-01-23

**Authors:** Johanna Wagner, Barbara Eiken, Imme Haubitz, Sven Lichthardt, Niels Matthes, Stefan Löb, Ingo Klein, Christoph-Thomas Germer, Armin Wiegering

**Affiliations:** 1 University Hospital Würzburg, Department of General, Visceral, Vascular and Pediatric Surgery, Würzburg, Germany; 2 Comprehensive Cancer Center Mainfranken, Core Unit Bioinformatics, Biocenter, University of Würzburg, Am Hubland, Würzburg, Germany; 3 Theodor Boveri Institute, Biocenter, University of Würzburg, Am Hubland, Würzburg, Germany; UKSH Campus Lübeck, GERMANY

## Abstract

**Background:**

Epidural catheters are state of the art for postoperative analgesic in abdominal surgery. Due to neurolysis it can lead to postoperative urinary tract retention (POUR), which leads to prolonged bladder catheterization, which has an increased risk for urinary tract infections (UTI). Our aim was to identify the current perioperative management of urinary catheters and, second, to identify the optimal time of suprapubic bladder catheter removal in regard to the removal of the epidural catheter.

**Methods:**

We sent a questionnaire to 102 German hospitals and analyzed the 83 received answers to evaluate the current handling of bladder drainage and epidural catheters. Then, we conducted a retrospective study including 501 patients, who received an epidural and suprapubic catheter after abdominal surgery at the University Hospital Würzburg. We divided the patients into three groups according to the point in time of suprapubic bladder drainage removal in regard to the removal of the epidural catheter and analyzed the onset of a UTI.

**Results:**

Our survey showed that in almost all hospitals (98.8%), patients received an epidural catheter and a bladder drainage after abdominal surgery. The point in time of urinary catheter removal was equally distributed between before, simultaneously and after the removal of the epidural catheter (respectively: ~28–29%). The retrospective study showed a catheter-associated UTI in 6.7%. Women were affected significantly more often than men (10,7% versus 2,5%, p<0.001). There was a non-significant trend to more UTIs when the suprapubic catheter was removed after the epidural catheter (before: 5.7%, after: 8.4%).

**Conclusion:**

The point in time of suprapubic bladder drainage removal in relation to the removal of the epidural catheter does not seem to correlate with the rate of UTIs. The current handling in Germany is inhomogeneous, so further studies to standardize treatment are recommended.

## Introduction

In Germany, approximately 16 million operations are performed every year, which include 2.5 million abdominal operations [1]. The implementation of the ERAS (enhanced recovery after surgery) concept in the early 1990s pursued the goal of an improved postoperative recovery with the help of an interdisciplinary team [2]. One key factor is the optimization of the postoperative analgesia. The epidural catheter is now the state of the art postoperative pain therapy [2,3]. Directly before the operation, an epidural catheter is placed in the epidural space. Analgesics are administered during the operation and typically for a few days after surgery [4]. The benefits of an epidural catheter for pain relieve is a reduction of gastrointestinal paralysis, nausea and vomiting [4]. On the other side, epidural administration of opioids mixed with a local anesthetic can lead to hypotension, itching and urinary retention [4]. High-dose epidurals using bupivacaine 0,25% have been shown to be associated with a rate for postoperative urinary tract retention (POUR) of up to 33% [5]. The risk for urinary tract retention leads to routine bladder catheterization, which itself is associated with a higher incidence of catheter-associated urinary tract infections (UTI) [6]. Catheter-related urinary tract infections are one of the most common nosocomial infections with a significant morbidity and costs [7]. 80% of nosocomial urinary tract infections are associated with the presence of urinary catheters [7]. A UTI is catheter-associated if the catheter was in place for more than 2 days or removed the day before the UTI appears [7]. With every day of transurethral catheterization, there is a 3–10% risk of bacteriuria [8,9]. One of the most important measures to prevent a catheter-associated UTI is to remove the catheter as soon as possible [10]. Suprapubic catheters are an alternative to urethral catheters with several advantages, such as patient comfort, less pain and better mobility [11]. Whether the risk for a catheter-associated UTI is lower in patients with suprapubic catheters compared to patients with a urethral catheter remains unclear [12]. A retrospective study comparing patients after rectum resection with a transurethral or suprapubic catheter showed similar infection rates (5.6 vs. 5.8%) [13]. Several studies have shown a slight decrease in the risk for a UTI, however, the quality of evidence is low, and the studies limited [14–16]. In contrast, other studies showed catheter-associated bacteriuria with suprapubic catheters in 95% of cases and UTIs in 11% [12].

In patients receiving a bladder catheter as well as an epidural catheter during abdominal surgery, the point in time of bladder catheter removal must be chosen wisely: not too early to induce complications of possible urinary retention due to the epidural catheter and not too late to induce urinary tract infections.

Up to date, no official recommendations exist as to when the urinary catheter should be removed during the postoperative period. Thus, the first aim of this study was to determine the current handling of bladder drainage and epidural catheters in the postoperative period after abdominal surgery in Germany. The second aim was to identify the optimal time frame of suprapubic bladder catheter removal in regard to the removal of the epidural catheter to reduce the risk for urinary tract infections.

## Methods

A questionnaire was sent to 102 German maximum care hospitals with a general and /or visceral surgery department (state and university hospitals). The questionnaire was sent to the chief surgeon’s office. We received 83 answers for the current handling of bladder drainage and epidural catheters. The questionnaire included the following questions:

- Do you use an epidural catheter in patients receiving abdominal surgery? YES / NO
- Do you apply a bladder drainage in patients receiving abdominal surgery? Transurethral or suprapubic catheter? YES / NO
- When do you remove the bladder drainage? Before removing the epidural catheter, simultaneously to the removal of the epidural catheter or after the removal of the epidural catheter? BEFORE / SIMULTANEOUSLY / AFTER
- Do you analyse the amount of residual urine before removing the bladder drainage? YES / NO And if so what is the amount of residual urine tolerated before removing the bladder drainage?

The answers were brought together anonymously in an Excel table and analysed with Excel Office 365.

Second, we conducted a retrospective single-center study including patients at the University Hospital Würzburg from 1^st^ of October 2012 to 1^st^ of August 2015. We included all patients age 15 to 89, who received an epidural catheter and a suprapubic bladder drainage after abdominal surgery (this included operations of the gastrointestinal tract, liver, gallbladder, pancreas, urogenital tract and peritoneum). The indication for an epidural anesthesia was an anticipated “major” abdominal surgery and did not include smaller operations, such as a laparoscopic cholecystectomy or appendectomy. We identified a total of 1250 patients, of which 501 patients were included in this study. 749 patients were excluded. Exclusion criteria were missing information on removal of the suprapubic catheter or the epidural catheter, incomplete data regarding urinary tract infections, exitus during the hospital stay, postoperative complications in need of additional surgery and postoperative sepsis. We divided the patients into three groups according to the point in time of suprapubic bladder drainage removal in regard to the removal of the epidural catheter (before, after and simultaneously). We then analyzed the onset of a urinary tract infection. A UTI was defined as the detection of over 10^5^ bacteria/ml. We defined the catheter-associated UTIs as those, which appeared in the first four days after removal of the last catheter and were diagnosed between the second and 14^th^ day after surgery. All other UTIs were defined as not catheter-associated. Due to the retrospective nature of the data and the lack of an established voiding test protocol, an analysis of urinary retention and voiding disorder postoperatively could not be analyzed with this dataset.

The data was analysed with a statistical software setup in Linux by an in-house biostatistician. Clinical parameters were compared with the Mann-Whitney U or Kruskal-Wallis test for continuous data and with the Fischer´s exact test for categorial variables. P < 0.05 was considered statistically significant. Multivariable analysis was by binary logistic regression. All variables with a p-value <0.1 in univariate analysis were included in the multivariate analysis.

## Results

### Results of the questionnaire

**Fig 1:**
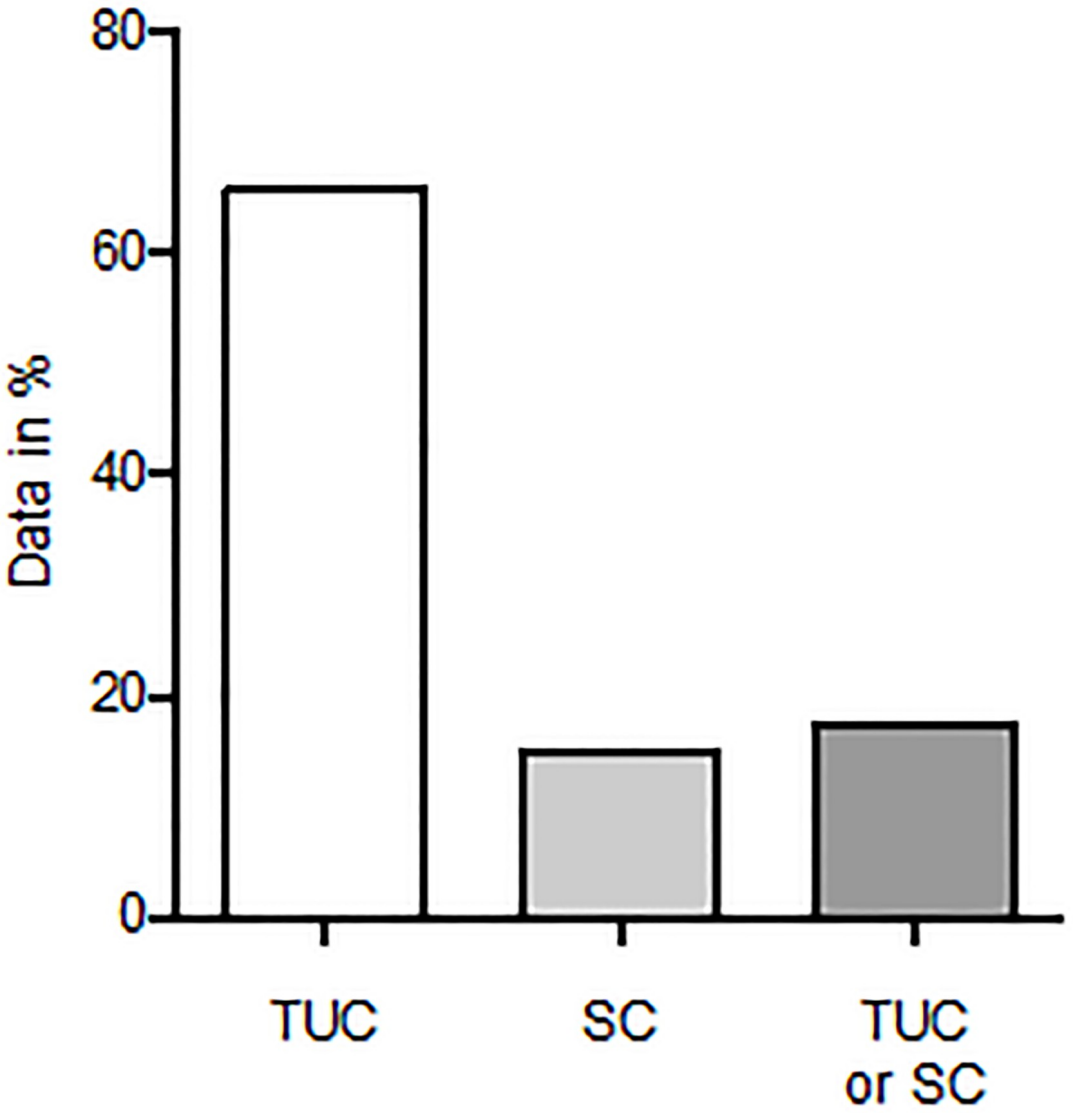
Type of urinary catheter used erioperatively after abdominal surgery. Most patients received a transurethral urinary catheter (TUC) postoperatively after abdominal surgery (men: 66.3%, women: 65.0%). 16.9% of men and 13.3% of women received a suprapubic catheter (SC).

**Fig 2:**
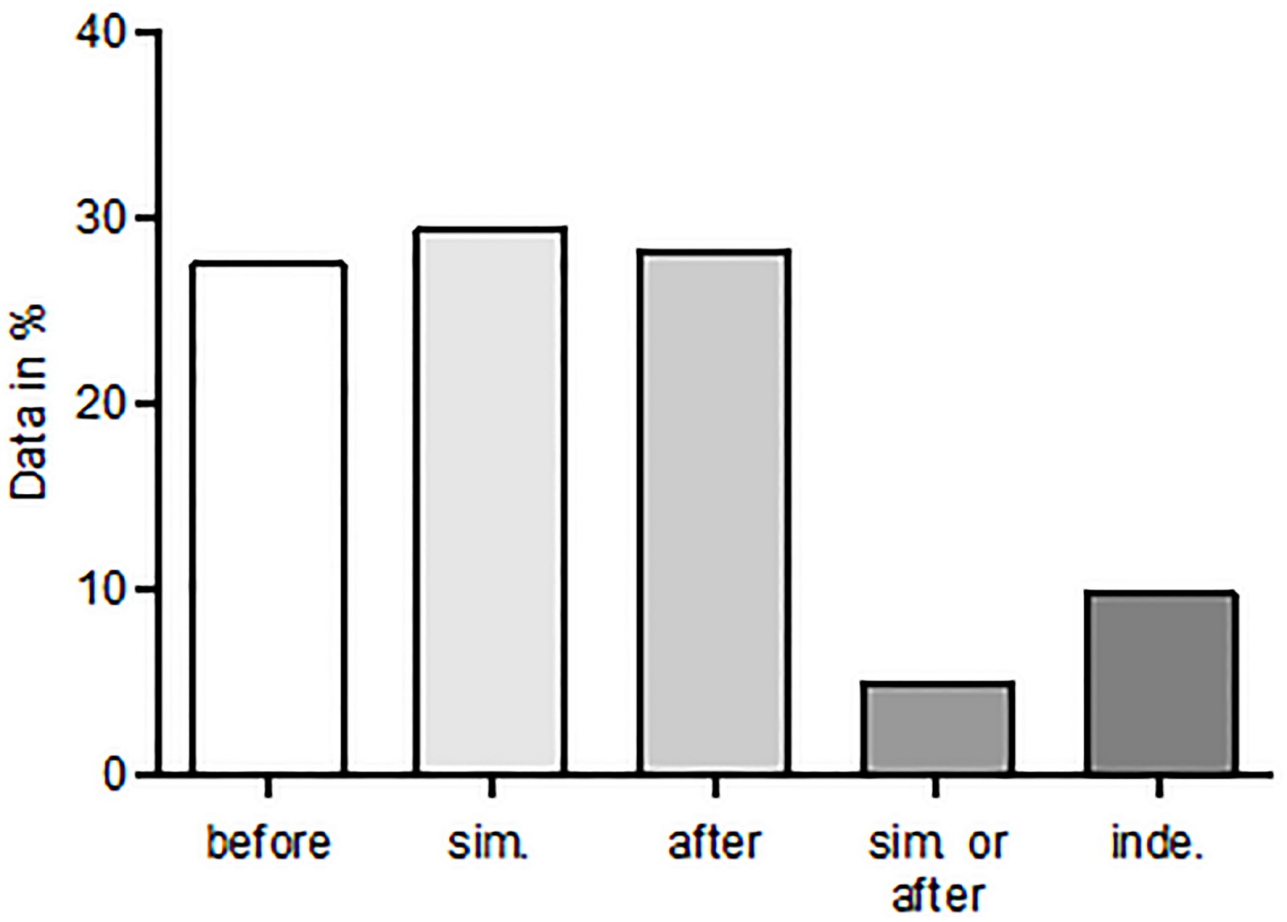
Point in time of urinary catheter removal in relation to the removal of the epidural catheter. The point in time of urinary catheter removal in relation to the removal of the epidural catheter was equally distributed among the three possible groups: before, simultaneously and after. sim. = simultaneously inde. = independent to removal of epidural catheter.

Out of the 102 questionnaires sent, there was a return rate of 81,4% (n = 83). In almost all hospitals (98.8%), patients received an epidural catheter for abdominal surgery and those patients also received a bladder drainage (98.8%, data not shown). The transurethral urinary catheter was used more often than the suprapubic catheter (men: 66.3% versus 16.9%; women: 65.0% versus 13.3%; see Fig 1). The point in time of urinary catheter removal in relation to the removal of the epidural catheter is not standardized. Thus, the removal of the urinary catheter is equally distributed between before, simultaneously and after the removal of the epidural catheter (see Fig 2). There was only a minor difference in the handling of male and female patients.

### Results of the retrospective study

**Fig 3:**
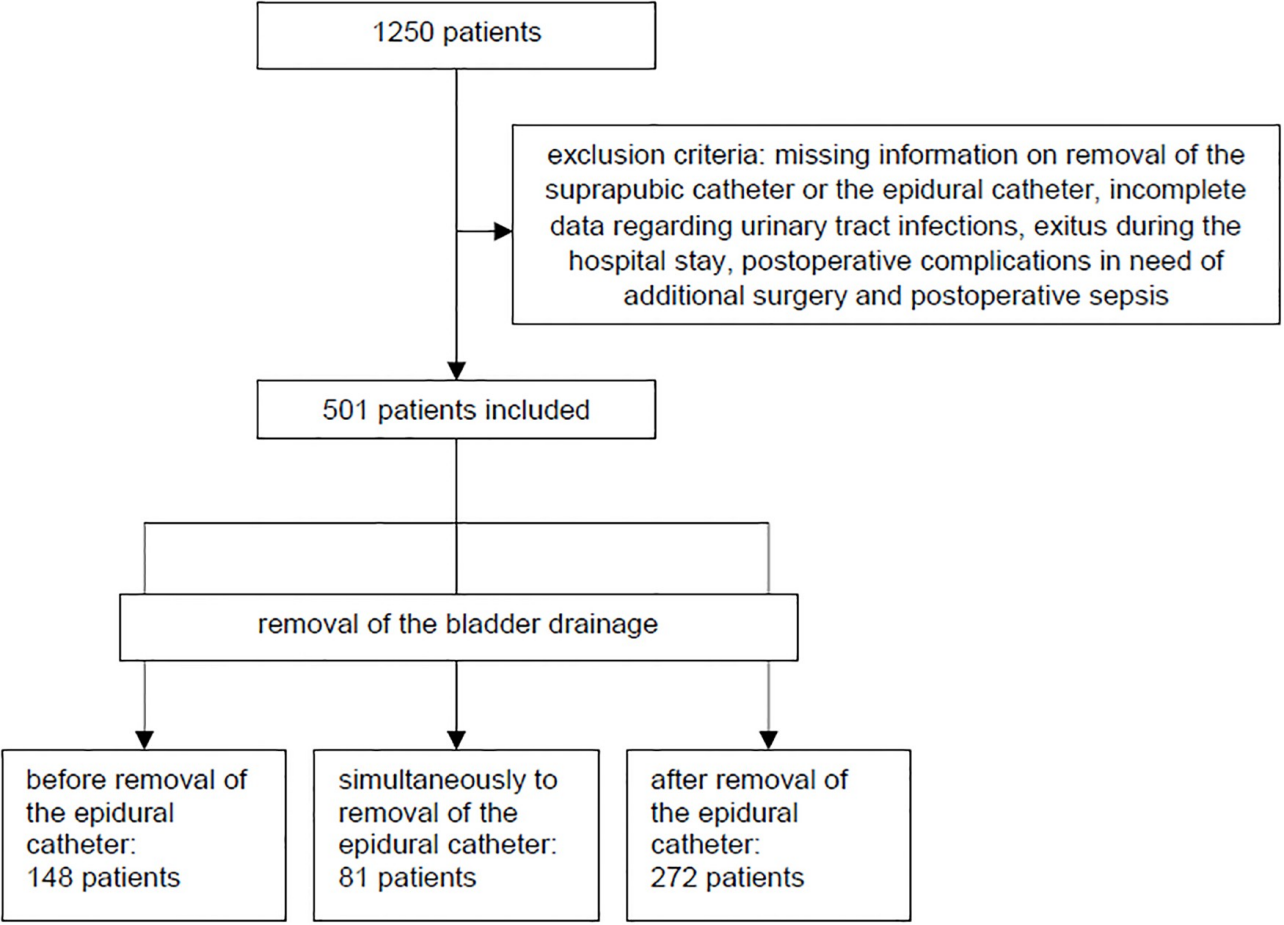
Study design.

Between 1^st^ of October 2012 and 1^st^ of August 2015, 1250 patients, aged 15 to 89, received an epidural catheter and a suprapubic bladder drainage after abdominal surgery at the University Hospital Würzburg. 749 patients were excluded due to our defined exclusion criteria, which was mainly due to missing information, leaving 501 patients, who were included in this study. The average age was 61.6 ± 14.2 years, 55% were male and 45% female (277 vs. 224 patients). In 148 patients (29.5%) the suprapubic bladder drainage was removed before the epidural catheter and in 272 patients (54.3%) after the removal of the epidural catheter. In 81 patients (16.2%) the suprapubic catheter and epidural catheter were removed on the same day (see Fig 3).

Tables 1 and 2 show the patient characteristics of the included 501 patients. The mean age was 61.6 ± 14.2 years. The Body Mass Index (BMI) measured between 15.6 and 43 kg/m^2^ with a mean BMI of 25.7 ± 4.5 kg/m^2^. The most common comorbidity was diabetes mellitus with 16.6%. Hepatobiliary and colon surgery were the two most common surgical procedures (32.7 and 37.9%). A differentiation between laparoscopic and open surgery was not made.

**Table 1:** Patient characteristics of all included patients.

Characteristic		mean
**Age**		61.6 ± 14.2 years
**BMI**		25.7 ± 4.5 kg/m^2^
**Duration of epidural catheterization**		4.7 ± 1.2 days
**Site of surgery**	Upper gastrointestinal tract	4.9 ± 1.3 days
	Hepatobiliary	4.9 ± 1.0 days
	Colon	4.5 ± 1.3 days
	Rectum	4.4 ± 1.5 days
	Peritoneal	5.4 ± 0.8 days
	Gynecological	4.3 ± 1.0 days
	Nephrological	5.0 ± 0.0 days
**Duration of suprapubic bladder catheterization**		6.1 ± 3.8 days
**Site of surgery**	Upper gastrointestinal tract	7.0 ± 4.3 days
	Hepatobiliary	6.5 ± 3.9 days
	Colon	5.2 ± 3.4 days
	Rectum	6.8 ± 4.0 days
	Peritoneal	6.1 ± 3.0 days
	Gynecological	4.3 ± 1.3 days
	Nephrological	8.0 ± 5.7 days
**Duration of the operation**		249.4 ± 115.1 mins

**Table 2:** Further patient characteristics of all included patients.

Characteristic		%	n
**Sex**	Male	55%	277
	Female	45%	224
**ASA Score** (American Society of Anesthesiologists)	I	3.8%	19
	II	62.3%	312
	III	32.9%	165
	IV	1.00%	5
**Comorbidities**	Diabetes mellitus	16.6%	83
	Cardiac concomitant diseases	14.4%	72
	Pulmonary concomitant diseases	10.4%	52
	Nephrological concomitant diseases	3.8%	19
	Chronic use of immunosuppressants	10.0%	50
**Site of surgery**	Upper gastrointestinal tract	11.0%	55
	Hepatobiliary	32.7%	164
	Colon	37.9%	190
	Rectum	14.4%	72
	Peritoneal	3.2%	16
	Gynecological	0.80%	4
	Nephrological	0.80%	4
**Point in time of suprapubic catheter removal**	Before removal of the epidural catheter	29.5%	148 81 272
	Simultaneously	16.2%	81
	After the removal of the epidural catheter	54.3%	272

#### Duration of suprapubic bladder and /or epidural catheterization

On average, the suprapubic catheter was removed 6.1 ± 3.8 days and the epidural catheter 4.7 ± 1.2 days after the operation (see Table 1). There was a positive correlation between the duration of suprapubic bladder catheterization and the age, ASA score and the length of the operation (see Table 3). Concerning the epidural catheter, there was a positive correlation between the BMI and the length of the operation (see Table 4).

**Table 3:** Correlation between duration of suprapubic bladder catheterization and age, ASA, BMI and operation length.

	n	tau	p
**Age**	501	0.0774	0.0096
**ASA**	501	0.1058	0.00040
**BMI**	501	0.0339	0.26
**Length of operation**	501	0.1953	<0.000005

**Table 4:** Correlation between duration of epidural catheterization and age, ASA, BMI and operations length.

	n	tau	p
**Age**	501	0.0381	0.20
**ASA**	501	0.0425	0.15
**BMI**	501	0.0912	0.0023
**Length of operation**	501	0.1820	<0.000005

#### Removal of the suprapubic bladder catheter in relation to the removal of the epidural catheter

The longer the duration of the operation, the later the bladder drainage was removed after the removal of the epidural catheter (tau = 0.13, p_τ_<0.001). In line with this correlation, the duration of operation was significantly longer in patients receiving the removal of the suprapubic catheter after the removal of the epidural catheter (266.1 ± 120.7 minutes versus 230.2 ± 109.3 minutes (before) and 228.7 ± 97.3 minutes (simultaneous removal), p_kw_ = 0.0019).

When regarding the different operations sites, the point in time of catheter removal differed. In patients receiving a colon or rectum resection the suprapubic bladder drainage was removed significantly more often after the removal of the epidural catheter (see Table 5). All other operation sites did not show a significant difference in the point in time of bladder drainage removal (data not shown, available in the supporting information file).

**Table 5:** Site of operation and comparison of point in time of catheter removal.

	n	%	n	%	
Removal of suprapubic catheter in regard to the removal of the epidural catheter	Colon resection				
	yes		no		
	(n = 186)		(n = 315)		
before	72	38.7%	76	24.1%	
simultaneously	32	17.2%	49	15.6%	
after	82	44.1%	190	60.3%	0.00083
	Rectum resection				
	yes		no		
	(n = 72)		(n = 429)		
before	12	16.7%	136	31.7%	
simultaneously	8	11.1%	73	17.0%	
after	52	72.2%	220	51.3%	0.0032

#### Occurrence of urinary tract infections

67 patients (13.4%) developed a urinary tract infection during the postoperative period (95%-CI: 10.6–16.7). 22 male patients (7.9%) and 45 female patients (20.1%) developed a postoperative urinary tract infection. This was significantly more often in females (p<0.001). 31 patients developed a catheter-associated urinary tract infection (6.7%, 95%-CI: 4.7–9.4).

36 patients of the 501 included patients received a transurethral bladder catheter after removal of the suprapubic catheter. Of those, 11 patients had a urinary tract infection (30.6%). Compared to the 465 patients with only a suprapubic catheter, significantly more patients with transurethral catheter had a UTI during the postoperative period (p_fy_ = 0.0042).

The length of the postoperative hospital stay was significantly longer in the patients with a UTI, whether the UTI was catheter-associated or not (no UTI: 14.6 ± 9.4 days; UTI, not catheter-associated: 20.3 ± 13.0 days; catheter-associated UTI: 20.5 ± 16.1 days; p_kw_<0.001).

We were then interested whether the occurrence of a UTI was associated with the point in time of the removal of the suprapubic bladder drainage or the removal of the epidural catheter. There was no significant difference in the occurrence of a UTI when the three patient groups were compared (bladder drainage removal before / simultaneously / after removal of the epidural catheter, see Table 6). In the multivariate regression we were able to determine the risk factors for a UTI: older age, female sex, longer duration of the operation. Patients with malignant diseases as primary diagnosis and reason for operation had significantly less UTIs (see Table 7). No significant difference in the UTI occurrence rate was seen when comparing the different operation sites (upper gastrointestinal tract: 1.8%, hepatobiliary 7.3%, colon 7.0%, rectum 5.6%, peritoneal 6.3%, gynecological and nephrological 0%; p = 0.8). The point of time of the removal of the suprapubic catheter did not influence the occurrence of UTIs but there was a tendency to an enhanced risk of UTI with a prolonged time of catheterization (see Table 6).

**Table 6:** Comparison of the three patient groups in regard to the occurrence of a UTI.

	**all UTIs**				
**Removal of the bladder drainage in relation to the removal of the epidural catheter**	yes		no		
	(n = 67)		(n = 434)		
	n	%	n	%	p
before	16	10.8%	132	89.2%	
simultaneously	9	11.1%	72	88.9%	
after	42	15.4%	230	84.6%	0.33
	**suprapubic catheter-associated UTIs**				
**Removal of the bladder drainage in relation to the removal of the epidural catheter**	yes		no		
	(n = 31)		(n = 434)		
	n	%	n	%	p
before	8	5.7%	132	94.3%	
simultaneously	2	2.7%	72	97.3%	
after	21	8.4%	230	91.6%	0.16

**Table 7:** Logistic regression for the occurrence of a UTI.

Predictor	n	Odds ratio	95%-CI		p(chi)
Basis	465				
Age	465	1.1	1.0	1.1	0.001
Sex (f)	465	6.1	2.5	15.0	0.000
Length of operation	465	1.0	1.0	1.0	0.004
Tumor operation	465	0.3	0.1	0.7	0.003
EC removal before SC removal	465	2.1	0.7	6.0	0.16

EC = epidural catheter

SC = suprapubic catheter

## Discussion

In our retrospective study, we were able to show that the point in time of removal of the suprapubic bladder catheter in relation to the removal of the epidural catheter does not influence the occurrence rate of urinary tract infections. The occurrence rate of UTIs was approximately identical in the three defined patient groups (removal of the bladder catheterization before / simultaneously / after the removal of the epidural catheter).

A UTI is catheter-associated if the catheter was in place for more than 2 days or removed the day before the UTI appears [14]. As we do not routinely screen for UTI after catheter removal, we defined the period of a catheter-associated UTI broader in order to register all possible catheter-associated UTIs. We suspect a latency of 1–2 days between first symptom and the realization of a urinalysis. The patient may already have the symptoms but time elapses until the nurse is notified and until the urinalysis is realized. Thus, we defined the catheter-associated UTIs as those, which appeared in the first four days after removal of the suprapubic catheter and were diagnosed between the second and 14^th^ day after surgery. With these criteria we detected catheter-associated UTIs in 6.67% of patients.

A few studies have examined the complications of the postoperative use of epidural catheters. It has been shown that use of an epidural catheter can lead to urinary retention, which then leads to prolonged bladder catheterization [5]. Prolonged bladder catheterization is associated with a higher incidence of catheter-associated UTIs [6]. Studies have shown that the intrathecal application of morphine can suppress bladder contraction and this effect can be reversed by the addition of naloxone [17,18]. In a prospective, randomized, double-blind study, Kim et al. showed that epidural sufentanil has less micturition problems as a side effect compared to epidural morphine. Thus, the authors suggest, the routine bladder catheterization may not be necessary beyond the first postoperative day [19]. In our study, in most patients (54.29%) the bladder catheter remained until after the epidural catheter was removed. The different reasons are not known, but one can assume that limited mobilization and urinary tract retention (measured by the amount of residual urine) are possible reasons for the prolonged bladder catheterization in our study. Interestingly, the rate of UTIs was not increased in this group, as one might expect in patients with prolonged bladder catheterization. With every day of transurethral catheterization, there is a 3–10% risk of bacteriuria [8,9]. One explanation for the missing increase in UTIs might be that the patients all received a suprapubic bladder drainage. Several studies have shown a slight decrease in the risk for a UTI in patients with a suprapubic bladder catheter in comparison to the transurethral bladder catheter. However, the quality of evidence is low and the studies limited [14,15]. Bonkat et al. showed catheter-associated bacteriuria with suprapubic catheters in 95% of cases and UTIs in 11% [12], which is comparable to the rates in patients with transurethral catheters [20] and with the data presented here. Bouchet-Doumenq et al. also showed comparable infection rates in patients with a suprapubic and transurethral bladder catheter [13]. In our study we were able to show an increased rate of UTIs in patients with a transurethral catheter when compared to those with a suprapubic bladder catheter (30.56 versus 12.04%, respectively; 6.67% suprapubic catheter-associated, p = 0.0042). However, this might by biased as these patients received a transurethral catheter after the suprapubic catheter was removed. The cause of re-catheterization is not known (urinary retention possible) and may be associated with occurrence of a UTI. Thus, a comparison of these two groups is not feasible.

Patients with rectum carcinoma undergoing rectum resection are a unique group of patients due to the additional risk of postoperative voiding dysfunction [21]. In a controlled study of patients after rectum resection the authors were able to show, that prolonged transurethral catheterization should be restricted to patients after resection of the lower rectum to avoid urinary tract retention. In all other patients (resection of the middle and upper rectum), the urinary catheter should be removed on the first postoperative day to avoid a urinary tract infection [22]. A retrospective study investigated the occurrence rate of urinary dysfunction after rectum resection and detected a rate of 38% [13]. In our study, patients with a rectum resection the suprapubic bladder drainage was removed significantly later. Interestingly, this patient group also did not show an increased rate of UTIs. Unfortunately, as no standardized protocol to detect urinary tract retention in our institution exists, we do not have any data on the frequency of urinary tract retention, particularly after rectum resection.

Due to the lack of national guidelines for the postoperative management of urinary catheters and epidural catheters in Germany, we conducted a nation-wide survey. In almost all German hospitals (98.80%), patients received an epidural catheter, as well as a bladder drainage after abdominal surgery. The transurethral urinary catheter was used more often than the suprapubic catheter (men: 66.27% versus 16.87%; women: 65.01% versus 13.25%). In our hospital we prefer the suprapubic catheter due to better patient comfort and the option of a voiding test before catheter removal to detect urinary tract retention. The insertion of the suprapubic catheter is more invasive than that of the transurethral catheter, but studies have shown comparable morbidity with both catheter types [13]. Due to the lack of evidence, the removal of the urinary catheter was equally distributed between before, simultaneously and after the removal of the epidural catheter.

Due to several limitations of this retrospective study, further investigations are necessary to determine the optimal point in time of bladder drainage removal in relation to the removal of the epidural catheter to reduce the risk of urinary tract infections.

In conclusion, guidelines do not exist, leading to an inhomogeneous postoperative protocol concerning the removal of bladder catheters. The point in time of removal of the suprapubic catheter in relation to the removal of the epidural catheter does not seem to influence the rate of catheter-associated UTIs. Nevertheless, the UTI rate seems to increase with length of bladder drainage. Due to a potentially reduced risk of UTI and an improved and increased mobilization, patients may benefit from an earlier removal of the suprapubic bladder catheter.

## Supporting information

S1 Table Point in time of suprapubic catheter removal in regard to the removal of the epidural catheter. (DOCX)

## References

[1] Statistisches Bundesamt (2016) Fallpauschalenbezogene Krankenhausstatistik (DRG-Statistik), Diagnosen und Prozeduren der vollstationären Patientinnen und Patienten in Krankenhäusern.

[2] Hoffmann H, Kettelhack C (2012) Fast-track surgery—conditions and challenges in postsurgical treatment. A review of elements of translational research in enhanced recovery after surgery. European surgical research. Europaische chirurgische Forschung. Recherches chirurgicales europeennes 49 (1): 24–34. 10.1159/000339859 22797672

[3] Hughes MJ, Ventham NT, McNally S, Harrison E, Wigmore S (2014) Analgesia after open abdominal surgery in the setting of enhanced recovery surgery. A systematic review and meta-analysis. JAMA surgery 149 (12): 1224–1230. 10.1001/jamasurg.2014.210 25317633

[4] Jorgensen H, Wetterslev J, Moiniche S, Dahl JB (2000) Epidural local anaesthetics versus opioid-based analgesic regimens on postoperative gastrointestinal paralysis, PONV and pain after abdominal surgery. The Cochrane database of systematic reviews (4): CD001893 10.1002/14651858.CD001893 11034732

[5] Hu Y, Craig SJ, Rowlingson JC, Morton SP, Thomas CJ, Persinger MB et al (2014) Early removal of urinary catheter after surgery requiring thoracic epidural. A prospective trial. Journal of cardiothoracic and vascular anesthesia 28 (5): 1302–1306. 10.1053/j.jvca.2014.05.009 25281046 PMC4185405

[6] Givens CD, Wenzel RP (1980) Catheter-associated urinary tract infections in surgical patients. A controlled study on the excess morbidity and costs. The Journal of urology 124 (5): 646–648. 7452793 10.1016/s0022-5347(17)55596-2

[7] Tenke P, Koves B, Johansen TEB (2014) An update on prevention and treatment of catheter-associated urinary tract infections. Current opinion in infectious diseases 27 (1): 102–107. 10.1097/QCO.0000000000000031 24345923

[8] Garibaldi RA, Mooney BR, Epstein BJ, Britt MR (1982) An evaluation of daily bacteriologic monitoring to identify preventable episodes of catheter-associated urinary tract infection. Infection control: IC 3 (6): 466–470. 6924646 10.1017/s0195941700056599

[9] Saint S, Lipsky BA, Goold SD (2002) Indwelling urinary catheters. A one-point restraint. Annals of internal medicine 137 (2): 125–127. 12118969 10.7326/0003-4819-137-2-200207160-00012

[10] Wald HL, Ma A, Bratzler DW, Kramer AM (2008) Indwelling urinary catheter use in the postoperative period. Analysis of the national surgical infection prevention project data. Archives of surgery (Chicago, Ill.: 1960) 143 (6): 551–557. 10.1001/archsurg.143.6.551 18559747

[11] Orikasa S, Kanbe K, Shirai S, Shintaku I, Kurosu S (2012) Suprapubic versus transurethral bladder drainage after radical prostatectomy. Impact on patient discomfort. International journal of urology: official journal of the Japanese Urological Association 19 (6): 587–590. 22404531 10.1111/j.1442-2042.2012.02980.x

[12] Bonkat G, Widmer AF, Rieken M, van der Merwe A, Braissant O, Muller G et al (2013) Microbial biofilm formation and catheter-associated bacteriuria in patients with suprapubic catheterisation. World journal of urology 31 (3): 565–571. 10.1007/s00345-012-0930-1 22926265

[13] Bouchet-Doumenq C, Lefevre JH, Bennis M, Chafai N, Tiret E, Parc Y (2016) Management of postoperative bladder emptying after proctectomy in men for rectal cancer. A retrospective study of 190 consecutive patients. International journal of colorectal disease 31 (3): 511–518. 10.1007/s00384-015-2471-8 26694925

[14] Tenke P, Kovacs B, Bjerklund Johansen TE, Matsumoto T, Tambyah PA, Naber KG (2008) European and Asian guidelines on management and prevention of catheter-associated urinary tract infections. International journal of antimicrobial agents 31 Suppl 1: S68–78. 18006279 10.1016/j.ijantimicag.2007.07.033

[15] Gould CV, Umscheid CA, Agarwal RK, Kuntz G, Pegues DA (2010) Guideline for prevention of catheter-associated urinary tract infections 2009. Infection control and hospital epidemiology 31 (4): 319–326. 10.1086/651091 20156062

[16] Branagan GW, Moran BJ (2002) Published evidence favors the use of suprapubic catheters in pelvic colorectal surgery. Diseases of the colon and rectum 45 (8): 1104–1108. 12195198 10.1007/s10350-004-6368-9

[17] Kuipers PW, Kamphuis ET, van Venrooij GE, van Roy JP, Ionescu TI, Knape JT et al (2004) Intrathecal opioids and lower urinary tract function. A urodynamic evaluation. Anesthesiology 100 (6): 1497–1503. 15166570 10.1097/00000542-200406000-00023

[18] Rawal N, Möllefors K, Axelsson K, Lingårdh G, Widman B (1983) An experimental study of urodynamic effects of epidural morphine and of naloxone reversal. Anesthesia and analgesia 62 (7): 641–647. 6859567

[19] Kim JY, Lee SJ, Koo BN, Noh SH, Kil HK, Kim HS et al (2006) The effect of epidural sufentanil in ropivacaine on urinary retention in patients undergoing gastrectomy. British journal of anaesthesia 97 (3): 414–418. 10.1093/bja/ael172 16816394

[20] Niël-Weise BS, van den Broek PJ (2005) Antibiotic policies for short-term catheter bladder drainage in adults. The Cochrane database of systematic reviews (3): CD005428 10.1002/14651858.CD005428 16034973

[21] Kwaan MR, Lee JT, Rothenberger DA, Melton GB, Madoff RD (2015) Early removal of urinary catheters after rectal surgery is associated with increased urinary retention. Diseases of the colon and rectum 58 (4): 401–405. 10.1097/DCR.0000000000000317 25751796

[22] Benoist S, Panis Y, Denet C, Mauvais F, Mariani P, Valleur P (1999) Optimal duration of urinary drainage after rectal resection. A randomized controlled trial. Surgery 125 (2): 135–141. 10026745

