# Supplementary material for: Suprapubic bladder drainage and epidural catheters following abdominal surgery—A risk for urinary tract infections?
Source: PLoS One. 2019 Jan 23;14(1):e0209825. doi: 10.1371/journal.pone.0209825 (PMC6343869; doi:10.1371/journal.pone.0209825)
Supplement: S1 Table — (DOCX) [file pone.0209825.s001.docx]

|  |  | **Point in time of suprapubic catheter removal in regard to the removal of the epidural catheter** | | | | | | | |
| --- | --- | --- | --- | --- | --- | --- | --- | --- | --- |
|  |  | before | |  | simultaneously | |  | after | |
| Operation site | n | | % | n | | % | n | | % |
| Upper gastrointestinal tract | 11 | | 20.0% | 6 | | 10.9% | 38 | | 69.1% |
| Hepatobiliary | 44 | | 26.8% | 31 | | 18.9% | 89 | | 54.3% |
| Colon | 72 | | 38.7% | 32 | | 17.2% | 82 | | 44.1% |
| Rectum | 12 | | 16.7% | 8 | | 11.1% | 52 | | 72.2% |
| Peritoneal | 6 | | 37.5% | 2 | | 12.5% | 8 | | 50.0% |
| Gynecological | 2 | | 50.0% | 1 | | 25.0% | 1 | | 25.0% |
| Nephrological | 1 | | 25.0% | 1 | | 25.0% | 2 | | 50.0% |
| Total | 148 | | 29.5% | 81 | | 16.2% | 272 | | 54.3% |
|  | | | | | | | | | |
